# Hyperprolactinemia in women: diagnostic approach

**DOI:** 10.61622/rbgo/2024FPS04

**Published:** 2024-04-25

**Authors:** Andrea Glezer, Heraldo Mendes Garmes, Leandro Kasuki, Manoel Martins, Paula Condé Lamparelli Elias, Vania dos Santos Nunes Nogueira, Ana Carolina Japur de Sá Rosa-e-Silva, Gustavo Arantes Rosa Maciel, Cristina Laguna Benetti-Pinto, Andrea Prestes Nácul

**Affiliations:** 1 Universidade de São Paulo Hospital das Clínicas Faculdade de Medicina São Paulo SP Brazil Hospital das Clínicas, Faculdade de Medicina, Universidade de São Paulo, São Paulo, SP, Brazil; 2 Universidade Estadual de Campinas Faculdade de Ciências Médicas Campinas SP Brazil Faculdade de Ciências Médicas, Universidade Estadual de Campinas, Campinas, SP, Brazil; 3 Universidade Federal do Rio de Janeiro Hospital Universitário Clementino Fraga Filho Rio de Janeiro RJ Brazil Hospital Universitário Clementino Fraga Filho, Universidade Federal do Rio de Janeiro, Rio de Janeiro, RJ, Brazil; 4 Universidade Federal do Ceará Departamento de Medicina Clínica e Núcleo de Pesquisa e Desenvolvimento de Medicamentos Fortaleza CE Brazil Departamento de Medicina Clínica e Núcleo de Pesquisa e Desenvolvimento de Medicamentos, Universidade Federal do Ceará, Fortaleza, CE, Brazil; 5 Universidade de São Paulo Faculdade de Medicina de Ribeirão Preto Departamento de Clínica Médica, Hospital das Clínicas Ribeirão Preto SP Brazil Departamento de Clínica Médica, Hospital das Clínicas, Faculdade de Medicina de Ribeirão Preto, Universidade de São Paulo, Ribeirão Preto, SP, Brazil; 6 Universidade Estadual Paulista Faculdade de Medicina de Botucatu Departamento de Clínica Médica Botucatu SP Brazil Departamento de Clínica Médica, Faculdade de Medicina de Botucatu, Universidade Estadual Paulista (UNESP), Botucatu, SP, Brazil; 7 Universidade de São Paulo Faculdade de Medicina de Ribeirão Preto Departamento de Ginecologia e Obstetrícia Ribeirão Preto SP Brazil Departamento de Ginecologia e Obstetrícia, Faculdade de Medicina de Ribeirão Preto, Universidade de São Paulo, Ribeirão Preto, SP, Brazil; 8 Universidade de São Paulo Departamento de Obstetrícia e Ginecologia Faculdade de Medicina Sao Paulo SP Brazil Departamento de Obstetrícia e Ginecologia, Disciplina de Ginecologia, Hospital das Clínicas HCFMUSP, Faculdade de Medicina, Universidade de São Paulo, Sao Paulo, SP, Brazil; 9 Universidade Estadual de Campinas Departamento de Obstetrícia e Ginecologia Faculdade de Ciências Médicas Campinas SP Brazil Departamento de Obstetrícia e Ginecologia, Faculdade de Ciências Médicas, Universidade Estadual de Campinas, Unicamp, Campinas, SP, Brazil; 10 Unidade de Reprodução Humana Hospital Fêmina Grupo Hospitalar Conceição Porto Alegre RS Brazil Unidade de Reprodução Humana, Hospital Fêmina, Grupo Hospitalar Conceição, Porto Alegre, RS, Brazil

## Keypoints

- Hyperprolactinemia (HPRL) is a cause of menstrual irregularity, galactorrhea, hypogonadism and infertility.
- Serum prolactin measurement should only be performed in the presence of compatible symptoms and/or in the presence of a pituitary tumor, even with an incidental diagnosis. Dosage is not recommended as a routine examination.
- There are several causes of HPRL. In most cases, it is caused by pregnancy, hypothalamic-pituitary disconnection or PRL-secreting pituitary adenomas (prolactinomas), or it can also be secondary to the use of medications.
- Recognizing clinical, laboratory and imaging findings is essential for the diagnosis of prolactinoma, and its differential diagnoses.

## Recommendations

- Hyperprolactinemia is a condition with diverse etiologies, and its correct identification is essential for the proper treatment and monitoring.
- Mild hyperprolactinemia should be confirmed with a new measurement after excluding venipuncture stress.
- Macroprolactin testing is indicated in patients with asymptomatic hyperprolactinemia.
- If drug-induced hyperprolactinemia is suspected, a new serum prolactin measurement is recommended three days after discontinuation of the drug, when withdrawal is possible. If there is a contraindication and doubt regarding the etiology, pituitary imaging should be performed.
- Sellar imaging by magnetic resonance, ideally, or computed tomography if the former is unavailable, should only be done after excluding other causes of HPRL. When sellar imaging is suggestive of a pituitary tumor, evaluate if the size of the lesion and prolactin levels point to the presumptive diagnosis of prolactinomas.

## Background

Increased serum prolactin (PRL) levels are a common finding when investigating patients with complaints of menstrual irregularity and infertility. Considering that appropriate evaluation of these patients is essential for correct management, experts from the Brazilian Society of Endocrinology and Metabolism (SBEM) and the Brazilian Federation of Gynecology and Obstetrics Associations (FEBRASGO) prepared this position to clarify the medical community on the main points of the management of hyperprolactinemia (HPRL).

Hyperprolactinemia is defined when serum PRL levels are higher than reference values for the normal population. These values are higher in women than in men, and generally less than 25 ng/mL. Reference values vary depending on the assay used.

A single PRL measurement is sufficient to establish the diagnosis in most cases. Given the pulsatile nature of this hormone’s release, a new dosage may sometimes be necessary in patients with mild HPRL. Prolactin release stimulation tests (e.g., after administration of hypothalamic thyrotrophin-releasing hormone / TRH) are not recommended.

Prolactin is the only pituitary hormone under negative tonus, by dopamine-mediated hypothalamic. It is the main hormone responsible for lactation in women and plays a role in regulating reproductive function by suppressing the gonadotropic axis. In most cases, HPRL is caused by pregnancy, hypothalamic-pituitary disconnection or PRL-secreting pituitary adenomas (prolactinomas), or it can also be secondary to drug use (Table 1). Regardless of the etiology, HPRL can cause hypogonadism, infertility and galactorrhea. After HPRL is confirmed, determining the etiology is essential for its appropriate management.

**Table 1:** Non-tumor causes of hyperprolactinemia

Physiological	Systemic	Pharmacological
Pregnancy	Chronic kidney disease – end stage	Antidepressants Neuroleptics/antipsychotics
Coitus	Polycystic ovary syndrome	Estrogens (oral contraceptives)
Breastfeeding	Cirrhosis	Antiemetics, antihistamines
Exercise	Chest wall disorders (surgery, shingles, piercing)	Antihypertensives, opioids
Stress	Primary hypothyroidism	Dopamine inhibitors

## What is the frequency of hyperprolactinemia in the general population and in subgroups of patients?

Hyperprolactinemia is the cause of amenorrhea in 10-20% of non-pregnant patients.^(1)^ In a study of 1,607 patients with clinically treated HPRL, the prevalence was approximately 10 per 100,000 in men and 30 per 100,000 in women, with a peak prevalence in women aged between 25-34 years.^(2^) Prolactinomas are the most common functioning pituitary adenomas, occurring with an annual incidence of approximately 30 per 100,000 inhabitants.^(3)^ In autopsy studies, the prevalence is much higher, since pituitary adenomas were identified in up to 11% of cases and almost half showed positive immunohistochemistry for PRL.^(4^)

## In which patients should prolactin be measured?

Serum PRL levels should only be measured in symptomatic patients, with symptoms such as galactorrhea, menstrual irregularity, infertility and decreased libido (Table 2). Dosing PRL as a routine check-up is not indicated. "Routine" dosing in asymptomatic patients can generate unnecessary costs and treatments (for example, HPRL due to macroprolactinemia in a patient with pituitary incidentaloma).

**Table 2:** Main signs and symptoms of hyperprolactinemia

Infertility
Oligo or amenorrhea
Galactorrhea
Hot flushes
Dyspareunia/vaginal dryness
Bone mass reduction
Reduced libido
Signs of tumor compression* (headache, reduced visual field, hypopituitarism)

* In patients with prolactinomas

Source: Adapted from Melmed et al. (2011).(5)

## What are common laboratory pitfalls in the evaluation of hyperprolactinemia?

The assessment of hyperprolactinemia of HPRL can be challenging without knowledge of its diagnostic pitfalls, leading to misdiagnosis and inappropriate treatment. Prolactin is usually measured by commercial two-site immunometric assays or sandwich principle immunoassays. The usual collection is carried out in the morning, after fasting for 2-3 hours. If there is no difficulty with venipuncture, a single collection is sufficient to determine the presence of HPRL.^(5,6)^ Mild HPRLs not consistent with the clinical presentation may be due to pulsatile PRL secretion. Exceptionally, a new confirmatory collection can be carried out with two samples at 15-20 minutes intervals.^(7,8)^ Two notable conditions regarding the prolactin dosing methodology must be considered, namely, the hook effect and macroprolactinemia.^(5,6)^

## Hook effect

The hook effect can occur when PRL is detected by sandwich assays. In this method, PRL binds to a capture antibody, generally stationary, and also to a signaling antibody, free in the supernatant, thus forming the "sandwich complex". After the incubation phase, excess unbound signaling antibodies are washed away, allowing the sandwich complex signal to be read. When PRL levels are excessively high, generally above 2,000 ng/mL, PRL binds to both antibodies, preventing complex formation, resulting in only moderately elevated concentrations of PRL.^(7,8)^ In cases of large tumors above 3 cm with PRL values between 30 and 120 ng/mL, successive dilutions of the serum are recommended to exclude the hook effect. This is particularly important for patients with pituitary macroadenomas and HPRL, where the PRL value will be the marker in distinguishing between a macroprolactinoma and a non-functioning pituitary macroadenoma with HPRL secondary to stalk disconnection, avoiding diagnostic and treatment errors.^(5,7,8)^ It is important to remember that PRL values above the detection range of the method must also be tested again with dilution to obtain the absolute PRL value.

## Macroprolactinemia

Macroprolactin is one of the three main forms of circulating PRL, together with the biologically active 23 kDa monomeric PRL and the dimeric PRL (big-PRL). Macroprolactin is a macromolecule composed of monomeric PRL coupled to high molecular weight IgG antibodies <150 kDa). Although biologically inactive, it can be detected in most commercial PRL assays. As macroprolactin has a high molecular weight, treating the sample with polyethylene glycol (PEG) will precipitate macroprolactin, allowing quantification of residual monomeric PRL in the supernatant. The residual PRL recovered after PEG treatment can be expressed as a percentage of the total pre-PEG PRL value. When macroprolactin is the predominant form, residual PRL after PEG is commonly less than 40%. Mild to moderate HPRL in an asymptomatic patient should lead to the determination of macroprolactinemia, avoiding unnecessary investigations and/or interventions.^(7)^

## What are the causes of hyperprolactinemia?

### Prolactinomas

Prolactinomas are tumors of lactotrophic cells and represent the main cause of pathological HPRL. Prevalence studies in several countries have demonstrated a prevalence of clinically relevant pituitary adenomas in approximately 1 case per 1,000 individuals, and prolactinomas as the most commonly diagnosed (53% of cases).^(9)^ In a Brazilian multicenter study involving 1,234 individuals with HPRL, prolactinomas accounted for 56.2% of cases.^(10)^ Prolactinomas are classified according to size into microadenomas (less than 10 mm in diameter) and macroadenomas (equal to or larger than 10 mm). The proportion between micro and macroprolactinomas in women has been reported as 8:1, and the peak age of occurrence is around 30 years.^(11)^ If no pituitary lesion is identified in patients with HPRL, and other causes have been ruled out, the patient is diagnosed with idiopathic HPRL. Although prolactinomas are mostly sporadic in cause, approximately 5% of cases may have a familial cause. They may be associated with multiple endocrine neoplasia type 1, isolated familial prolactinomas or familial pituitary tumors.^(12)^ Prolactinoma should be investigated in women with HPRL in whom physiological, pharmacological causes and chronic diseases that could cause this hormonal change have been ruled out. Prolactinoma should also be investigated in patients with pituitary adenoma, even if they deny complaints related to hypogonadism. Macroprolactinomas should be considered in any patient with neurological or ophthalmological signs and symptoms resulting from mass effect in the sellar region, such as headache or visual field changes with associated hypopituitarism.^(13)^ Since a significant number of patients with acromegaly may also present HPRL, it is important to measure PRL in these patients.^(5)^ This HPRL may be the result of a pituitary adenoma cosecreting PRL and growth hormone (GH),^(14)^ as well as the compression of the pituitary stalk. Compared to patients with a purely GH-secreting adenoma, acromegalic patients with HPRL present an earlier onset of the disease, but with less expressive physical characteristics of acromegaly.^(15)^ Therefore, whenever possible, we recommend screening patients with prolactinoma for acromegaly at the time of diagnosis by measuring insulin-like growth factor type 1 (IGF-1) without the need for routine measurements in the follow-up of these individuals.

## When prolactinoma is excluded, what other causes should be investigated?

The main non-tumorous cause of HPRL is secondary to drugs. The topic will be covered in the following section. Other non-tumor causes of HPRL include physiological causes, systemic diseases (Table 1) and diseases of the hypothalamic-pituitary region.^(5,16)^ The most common physiological causes are: pregnancy and breastfeeding, coitus, nipple manipulation and physical exercise. Except during pregnancy and breastfeeding, in which HPRL can reach values above 200 ng/mL, the elevation of PRL in other situations is slight, rarely leading to galactorrhea or menstrual irregularity.^(17,18)^ Systemic diseases can also lead to HPRL through different mechanisms. In end-stage chronic kidney disease, decreased clearance is the main factor in the elevation of PRL.^(19)^ Alterations in the nerve endings of the chest wall lead to decreased inhibition by dopamine,^(20^) also observed in liver cirrhosis, which also presents increased estrogen concentrations.^(21^) Hyperprolactinemia is found in 20-40% of patients with primary hypothyroidism and is caused by the elevation of TRH, a lactotroph secretagogue. The elevation of PRL appears to be directly related to TSH concentrations, which is a mandatory test in the differential diagnosis of HPRLs. With the treatment of hypothyroidism, PRL normalizes.^(22,23)^ The prevalence of HPRL in patients with polycystic ovary syndrome (PCOS) is quite variable. Therefore, HPRL associated with PCOS should be a diagnosis of exclusion. Notably, PRL values above 60-80 ng/mL suggest another underlying cause of HPRL that should be actively investigated. Hypothalamic-pituitary diseases comprise several entities, including neoplastic, granulomatous, infectious and infiltrative diseases, and can cause the pituitary stalk disconnection either by sectioning, compressing or stretching it (Table 3). Consequently, there will be a decrease in the dopaminergic inhibitory tone on PRL secretion, resulting in HPRL. Hyperprolactinemia is typically more pronounced with obvious symptoms (galactorrhea, infertility and hypogonadism), but it rarely exceeds 100 ng/mLs. Therefore, in the case of a patient with persistent HPRL, when other etiologies having been ruled out, it is recommended to perform an imaging examination of the sella turcica, preferably Magnetic Resonance Imaging (MRI) or computed tomography, when MRI is not available. The differential diagnosis of HPRL is of paramount importance, since in many of these etiologies, specific treatment, other than dopaminergic agonists (DAs), is indicated. Starting DA treatment for HPRL without adequate investigation will delay the diagnosis of the underlying disease with possible disastrous consequences.

**Table 3:** Conditions of the hypothalamic-pituitary region that can lead to hyperprolactinemia due to stalk disconnection (except for prolactinomas)

Tumors	Infiltrative	Inflammatory	Others
Prolactinomas Pituitary macroadenomas (non-functioning, secreting, Nelson’s syndrome *)	Langerhans cell histiocytosis	Lymphocytic hypophysitis	Rathke’s pouch cyst
Craniopharyngioma	Sarcoidosis	IgG4related hypophysitis	Section of the pituitary stalk (trauma)
Metastases (breast, lung)	Tuberculosis	Hypophysitis in granulomatosis with polyangiitis	Internal carotid artery aneurysm
Germ cell tumors			Empty sella
Others			Radiotherapy
			Idiopahtic

* Nelson’s syndrome: growth of corticotropinoma with significant elevation of serum ACTH levels, which may occur after bilateral adrenalectomy

In cases of positive imaging, the etiology of HPRL can be defined by evaluating the correlation between HPRL and the size of the pituitary lesion. Most authors agree that in the presence of an image suggestive of pituitary macroadenoma, PRL levels above 200 ng/mL and the absence of other causes for HPRL, the diagnosis of prolactinomas is suggested, whereas if levels are below 100 ng/mL, HPRL by stalk disconnection is suggested.^(5,26)^

However, in some cases of pituitary adenoma, doubt about the etiological diagnosis may persist. Prolactin levels in pituitary stalk disconnection caused by macroadenomas and small prolactinomas may overlap. Pituitary incidentalomas are common, with an estimated global prevalence of 16.7%.^(28)^ Therefore, in the case of a small, non-functioning pituitary adenoma and low PRL concentrations, the initial differentiation between microprolactinoma and non-functioning incidentaloma is not as clear. Only evolution with tumor reduction after treatment with DA can confirm the diagnosis of prolactinoma, as it is known that non-functioning microadenomas increase in size in approximately 10-20% of cases or remain stable,^(29,30)^ while microprolactinomas regress in size or disappear after DA therapy in the vast majority of cases. Note that in surgical series of pituitary microadenomas considered as prolactinomas in the preoperative period, approximately 17% did not confirm the diagnosis of prolactinomas by immunohistochemistry, showing that they were non-functioning lesions erroneously diagnosed as prolactinomas due to stalk disconnection.^(31)^ These data reinforce the importance of monitoring tumor size after DA prescription for later differential diagnosis between prolactinomas or non-functioning lesions of the pituitary region that induced an increase in serum PRL levels due to disconnection of the pituitary stalk.

## How to manage drug-induced hyperprolactinemia?

Drugs are a frequent cause of HPRL. The global estimate is that medication-induced HPRL can vary from 15% to 45%.^(10,32,33)^ Many medications can cause different degrees of HPRL; antipsychotics are the most frequently associated with HPRL, particularly first-generation ones. Currently, there are several therapeutic options that can be used with less effect on PRL concentrations (Table 4).

**Table 4:** Medications that cause hyperprolactinemia

**Antipsychotics**	
Chlorpromazine/thioridazine/levomepromazine	+++
Haloperidol	+++
Sulpiride/tiapride	+++
Risperidone	+++
Quetiapine	+
Olanzapine	+
Pimozide	+
Clozapine	0
Aripiprazole	0

**Antidepressants**	
Clomipramine	+++
Amitriptyline	+
Citalopram	±
Fluvoxamine	±
Paroxetine	CR
Fluoxetine	CR
Imipramine	0
Bupropion	0
Nortriptyline	0
Sertraline	0
Trazodone	0

**MAOI**	
Pargyline	+++
Clorgyline	+
Tranylcypromine	±

**Anti-hypertensive**	
Reserpine	++
Methyldopa	+
Verapamil	+
Labetalol	+

**Gastrointestinal**	
Domperidone/metroclopramide	+++
Cimetidine/ranitidine	+

**Anorectics**	
Fenfluramine/amphetamines	+

**Opiates and cocaine**	+

**Protease inhibitors**	+

**Estrogen**	+

* CR - Relato de casos isolados; 0 - no effect;

± - minimal increase, but not at abnormal levels;

+ - increase in abnormal levels in a small percentage of patients;

++ - increase in abnormal levels in 25-50% of patients;

+++ - increase in abnormal levels in more than 50% of patients, reaching values >200 ng/mL;

MAOI - monoamine oxidase inhibitors

Source: Adaptea from Molitch (2008).^(34^)

The mechanisms by which drugs lead to HPRL are: inhibition of dopamine by antagonistic action of these substances on dopamine receptors (antipsychotics and metoclopramide) or inhibition of dopamine synthesis (estrogen).^(32)^ Other substances can act on the secretion of factors that alter the tonic suppression of PRL synthesis such as serotonin and GABA. These mechanisms affect the inhibitory control of dopamine. From a practical point of view, in most drug-induced HPRLs, PRL concentrations are less than 100 ng/mL.^(35)^ However, the PRL concentration alone does not guarantee that the cause is solely medication-related.^(35)^ The differential diagnosis with organic causes is one of the first steps in the investigation.^(4,8)^ Drug-induced HPRL can cause the same symptoms as those caused by PRL-producing tumors, which can lead to menstrual irregularities, galactorrhea and infertility.^(4,10)^ Therefore, some strategies are adopted to differentiate the two causes: new PRL dosage after temporarily stopping the medication or changing the medication to another that does not alter the PRL; or investigation using imaging methods when there is no possibility of withdrawal/change of medication. Note that the withdraw or change of medication should always be done in conjunction with the psychiatrist or the prescriber of the basic medications. An additional suggestion is to investigate patients with PRL levels greater than 100 ng/mL who use medications known to increase PRL with imaging methods.^(35)^ Once tumor causes have been excluded, especially macroadenomas, if it is impossible to change the medication, the prescription of hormonal therapies containing estrogen associated with progestogen, should be considered in women with a uterus to minimize the consequences of hypoestrogenism. When contraception is necessary, consider combined hormonal contraceptives.

## What changes regarding the diagnosis of hyperprolactinemia after menopause?

Hyperprolactinemia is usually diagnosed in young women during menacme. There is little evidence of how hormonal changes resulting from menopause interfere with PRL production. As postmenopausal women are amenorrheic, the classic complaint of menstrual irregularity due to HPRL is not noticed, which delays the diagnosis of pituitary adenomas and makes the real incidence of prolactinomas at this stage of life unknown. Microadenomas are rarely diagnosed after menopause. Most diagnoses in this age group are larger tumors often investigated due to the presence of compressive symptoms (visual changes and headache) resulting from mass effect that may also present parasellar extension. A multicenter study including 14 women with macroadenomas diagnosed after menopause showed that six of them had visual changes, while galactorrhea was a symptom reported by only three.^(36)^ Prolactin concentrations at diagnosis were quite high and even with late diagnosis, these authors demonstrated a good therapeutic response to DA. There is little data in the literature on prolactinomas diagnosed during menopause,^(37)^ and 92% of cases were macroprolactinomas.

**Figure 1:**
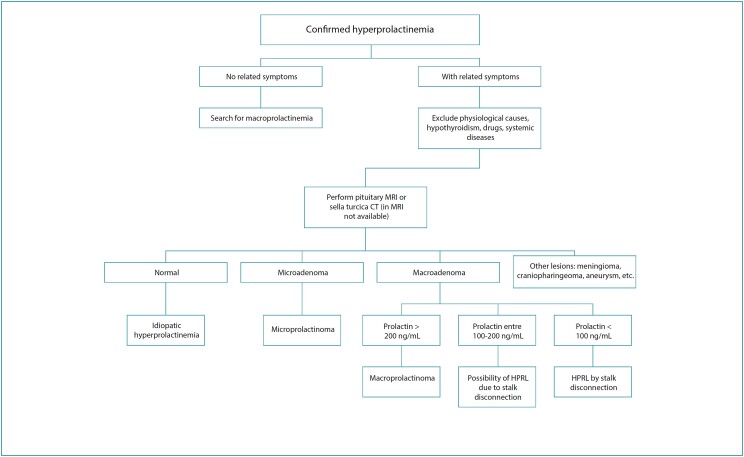
Flowchart of diagnostic assessment of hyperprolactinemia

In summary, we suggest the following flowchart for diagnosing HPRL (Figure 1).

## Final considerations

This position statement was prepared jointly by the Department of Neuroendocrinology of the Brazilian Society of Endocrinology and Metabolism (SBEM) and the National Specialized Commission on Gynecological Endocrinology of the Brazilian Federation of Gynecology and Obstetrics Associations (FEBRASGO). The aim of this document is to provide updated information to assist gynecologists, endocrinologists, and primary care physicians in diagnosing hyperprolactinemia in women.

## National Specialized Commission on Endocrine Gynecology of the Brazilian Federation of Gynecology and Obstetrics Associations (FEBRASGO)

### President:

Cristina Laguna Benetti Pinto

### Vice president:

Ana Carolina Japur de Sa Rosa e Silva

### Secretary:

José Maria Soares Júnior

### Members:

Andrea Prestes Nácul

Daniela Angerame Yela

Fernando Marcos dos Reis

Gabriela Pravatta Rezende

Gustavo Arantes Rosa Maciel

Gustavo Mafaldo Soares

Laura Olinda Rezende Bregieiro Costa

Lia Cruz Vaz da Costa Damásio

Maria Candida Pinheiro Baracat Rezende

Sebastião Freitas de Medeiros

Tecia Maria de Oliveira Maranhão

Vinicius Medina Lopes
